# A Comprehensive Exploration of the Multifaceted Neuroprotective Role of Cannabinoids in Alzheimer’s Disease across a Decade of Research

**DOI:** 10.3390/ijms25168630

**Published:** 2024-08-07

**Authors:** Petros Tyrakis, Christina Agridi, Malamati Kourti

**Affiliations:** Department of Life Sciences, School of Sciences, European University Cyprus, Nicosia 1516, Cyprus; petrostyrakis3@gmail.com (P.T.); chr.agridi@gmail.com (C.A.)

**Keywords:** cannabinoids, Alzheimer’s disease, *Cannabis sativa*, CB_1_R, CB_2_R, Trpv-1, GPR-55

## Abstract

Alzheimer’s disease (AD), a progressive neurodegenerative disorder, manifests through dysregulation of brain function and subsequent loss of bodily control, attributed to β-amyloid plaque deposition and TAU protein hyperphosphorylation and aggregation, leading to neuronal death. Concurrently, similar cannabinoids to the ones derived from *Cannabis sativa* are present in the endocannabinoid system, acting through receptors CB_1_R and CB_2_R and other related receptors such as Trpv-1 and GPR-55, and are being extensively investigated for AD therapy. Given the limited efficacy and adverse effects of current available treatments, alternative approaches are crucial. Therefore, this review aims to identify effective natural and synthetic cannabinoids and elucidate their beneficial actions for AD treatment. PubMed and Scopus databases were queried (2014–2024) using keywords such as “Alzheimer’s disease” and “cannabinoids”. The majority of natural (Δ^9^-THC, CBD, AEA, etc.) and synthetic (JWH-133, WIN55,212-2, CP55-940, etc.) cannabinoids included showed promise in improving memory, cognition, and behavioral symptoms, potentially via pathways involving antioxidant effects of selective CB_1_R agonists (such as the BDNF/TrkB/Akt pathway) and immunomodulatory effects of selective CB_2_R agonists (TLR4/NF-κB p65 pathway). Combining anticholinesterase properties with a cannabinoid moiety may enhance therapeutic responses, addressing cholinergic deficits of AD brains. Thus, the positive outcomes of the vast majority of studies discussed support further advancing cannabinoids in clinical trials for AD treatment.

## 1. Introduction

Alzheimer’s disease (AD) is a neurodegenerative disorder of acquired dementia, which progressively destroys a significant part of the brain’s neuronal network. This disease belongs to the broad spectrum of dementia disorders, with the latest data classifying it as a neurocognitive disorder [1]. Today, AD is responsible for over 50% of dementia diagnoses, associated with dysfunctions in memory, cognitive and motor skills, speech, senses, visuospatial abilities, and attentional focus [2]. These symptoms bring about behavior alterations in AD patients, which in turn will lead to serious impairment of their social and/or professional abilities and lives. Consequentially, three main stages of disease progression are observed: the early (preclinical), which is devoid of symptoms (and can be diagnosed by the analysis of mainly protein biomarkers), the intermediate, where the first symptoms appear in the form of mild cognitive impairment, and the final stage, leading to the conclusive diagnosis of AD [3].

Alzheimer’s disease is primarily characterized by two key neuroanatomical changes: senile neuritic plaques and neurofibrillary tangles (NFTs), resulting from abnormal protein formations. Neuritic plaques form due to the abnormal deposition of beta-amyloid (Aβ) protein, particularly Aβ oligomers, known for their neurotoxicity. Aβ, mainly found in the hippocampus and entorhinal cortex, disrupts memory and learning processes, with synapses being initial targets. Other frequently affected areas include the amygdala and the frontal, temporal, and parietal lobes. Conversely, amyloid plaques are less frequently found in the occipital cortex and cerebellum [4]. Additional neuroanatomical changes in AD include cortical thinning, particularly in the temporal and parietal regions, neurofibrillary tangles, which comprise abnormal accumulations of TAU protein inside neurons affecting the hippocampus and spreading to neocortical areas, hippocampal atrophy, which affects memory and learning, white matter changes including reduced integrity of white matter tracts, and synaptic loss, leading to malfunctioning communication within neural networks [5,6,7]. All these changes are accompanied by chronic, low-grade neuroinflammation [8].

Genetic factors play a significant role, with overexpression of the amyloid precursor protein (*APP*) gene linked to the disease, notably seen in trisomy 21 (Down syndrome) patients. Mutations in the *APP* gene lead to increased production of Aβ proteins, contributing to early-onset familial AD [5,9]. The pathogenesis of AD involves two metabolic pathways of APP: the non-amyloidogenic and amyloidogenic pathways. Imbalance towards the latter leads to increased production of insoluble Aβ monomers, promoting plaque formation. Dysfunctions in Aβ clearance enzymes, such as apolipoprotein E (apoE), further exacerbate this imbalance [10]. Other genetic variations involved include *apoE ε4*, presenilin 1 and 2 (*PSEN1* and *PSEN2*), ATP binding cassette subfamily A member 7 (*ABCA7*), *CD33*, phospholipase D3 (*PLD3*), *clusterin*, bridging integrator 1 (*BIN1*), sortilin-related receptor 1 (*SORL1*), and triggering receptor expressed on myeloid cells 2 (*TREM2*), to name a few [11]. Microglia and other cells attracted to the area may cause further plaque formation from the amorphous fragments of degenerated neuronal cells, subsequently releasing pro-inflammatory molecules and inducing oxidative stress [12,13]. Aβ aggregation triggers neurotoxic phenomena, including disruption of calcium homeostasis and hyperphosphorylation of the TAU protein, intensifying neurotoxic effects [10,14]. Hyperphosphorylated TAU protein, enhanced by decreased expression of certain phosphatases such as protein phosphatase 2A (PP2A) and protein phosphatase 1 (PP1), aberrant activation of kinases such as glycogen synthase kinase-3 beta (GSK-3β), chronic stress, and other diseases [15,16,17], forms insoluble filaments (paired helical filaments, PHFs), leading to NFTs and neuronal atrophy [18]. Although the decreased expression of the abovementioned phosphatases has been recently viewed with criticism [19,20], the rest of the factors continue to play a significant role. Brain changes in AD include temporal lobe degeneration, especially in the hippocampus, affecting memory formation. Progressive neurodegeneration results in cortical atrophy, particularly in posterior regions. Anatomical changes manifest as cerebral sulci enlargement and lateral ventricle expansion [21,22].

The most common methods for AD management currently available include a few basic classes of drugs. These classes mainly include acetylcholinesterase (AChE) inhibitors such as rivastigmine, donepezil, and galantamine, as well as inhibitors of activated NMDA receptors, such as memantine [23]. AChE inhibitors enhance cholinergic neurotransmission, which is considered to have a key role in memory and learning functions, while memantine mitigates excitotoxicity, which has been associated with neuronal death, thereby normalizing glutamate neurotransmission [24]. Brexpiprazole belongs to the atypical antipsychotics and is the only approved drug against the characteristic agitation symptoms of AD, such as akathisia, extreme verbal and physical behavior, repetitive movements, etc. [25]. These pharmacotherapies may offer temporary relief from AD symptoms. However, none of them provide a cure, as they cannot halt the progression of the disease, instead providing only symptomatic treatment [23].

The U.S. Food and Drug Administration (FDA) has already approved the use of three disease-modifying immunotherapies, donanemab, lecanemab, and aducanumab, which are anti-amyloid monoclonal antibodies that minimize the total number of Aβ plaques and seem promising for curing cognitive impairment if taken in the early stages of AD. Nevertheless, they are accompanied by severe adverse effects, including brain swelling and bleeding, severe allergic reactions, etc. [26]. Alternative treatment options are urgently needed. 

## 2. Endocannabinoid System and Its Association with Alzheimer’s Disease

Cannabinoids are the main components that can be isolated from hemp or marijuana, i.e., from the dried leaves and flowers of the plant species *Cannabis sativa* [27]. This plant product is considered one of the most widely used illicit drugs worldwide [28] and contains two characteristic cannabinoids, tetrahydrocannabinol (Δ^9^-THC) and cannabidiol (CBD). Its consumption is followed by psychotropic effects, due to its action on the central nervous system (CNS), for which Δ^9^-THC is responsible. CBD lacks psychotropic properties and is mainly responsible for the relaxing effects of marijuana [29]. Cannabinoids show affinity with receptors in the central and peripheral nervous system (PNS). These receptors, in combination with cannabinoids that occur naturally in the body (endocannabinoids), make up the endocannabinoid system (ECS). 

The ECS is a sophisticated signaling network involving cannabinoid receptors, endogenous ligands (endocannabinoids), and a range of biosynthetic and hydrolytic enzymes. In particular, CB_1_ (CB_1_R) and CB_2_ (CB_2_R) receptors are reported, with CB_1_Rs normally found in CNS regions such as the hippocampus, cerebellum, and basal ganglia neurons, as well as in tissues innervated by the PNS such as the heart and liver and also in cells of the immune system. CB_2_Rs mainly exist in peripheral tissues, such as cells of the immune system, including monocytes, macrophages, B cells, and T cells. In the nervous system, CB_2_Rs are primarily located in microglia and astrocytes, where they play a role in responding to various damaging conditions associated with local inflammation [30]. Additionally, CB_2_Rs are expressed in specific types of neurons, including hippocampal neurons and dorsal root ganglion neurons, although their expression in neurons is generally more limited compared to microglia [31,32]. CB_1_R and CB_2_R belong to the class of G-protein-coupled receptors (GPCR), whose role is the inhibitory regulation of neurotransmitters such as glutamate and G-aminobutyric acid (GABA) [33,34], with CB_1_Rs being solely responsible for the psychotropic effects of cannabinoids.

The ECS plays a crucial physiological role, encompassing functions like inflammation and immune regulation, promotion of apoptotic processes, stimulation of neurogenesis, nociception, and the display of antioxidant properties [32,35]. Anandamide (AEA), 2-arachidonylglycerol (2-AG), and 2-arachidonyl glyceryl ether or nolantine ether (2-AGE) are the endocannabinoids constituting this system, functioning as lipid retrograde neurotransmitters that work through cannabinoid receptors [36]. They are synthesized by enzymes such as N-acylphosphatidylethanolamine phospholipase D (NAPE-PLD) and diacylglycerol lipase (DAGL), respectively. These substances participate in a myriad of biological processes, governing essential functions including memory, learning, neuronal development, emotional regulation, sleep, temperature control, pain modulation, appetite regulation, hormonal balance, and immune system regulation, including inflammation [37]. The degradation of endocannabinoids is primarily mediated by fatty acid amide hydrolase (FAAH) and monoacylglycerol lipase (MAGL), ensuring rapid termination of their signaling activities [38].

In addition to the classical cannabinoid receptors, endocannabinoids also interact with non-classical receptors such as Trpv-1, which is involved in pain and inflammation, and GPR-55, which contributes to neuroprotection and regulation of the immune response. Activation of these receptors influences multiple signaling pathways, including mitogen-activated protein kinase (MAPK) and phosphatidylinositol-3-kinase (PI3K), highlighting the ECS’s role in synaptic plasticity, cell migration, and neuroprotection [38].

### 2.1. Neuropathological Evidence for Endocannabinoid System Involvement in Alzheimer’s Disease

Animal experiments have revealed the protective role of CB_1_Rs against AD-related pathologies [33,39]. These receptors are pivotal in fundamental brain functions such as cognition, memory, emotion, motor control, hunger, and pain sensation [40], as well as in regulating energy balance and metabolism [41]. During the progression of AD, there is a gradual dysfunction of the ECS [42]. This is marked by alterations in the levels and/or expression areas of both CB_1_R and CB_2_R. Initially, CB_1_R expression increases in the frontal cortex and hippocampus during the early stages of AD, but decreases progressively over time. Conversely, CB_2_R expression becomes exclusive to microglial cells and elevates notably, likely due to intense neuroinflammation, in the later stages of AD [33]. At the same time, the levels of AEA in cortical areas decrease, directly correlating with cognitive decline [36]. All these changes highlight the extensive involvement of CBRs in neurodegenerative disease-linked biological processes, emphasizing their importance in addressing AD pathology [37].

### 2.2. Endocannabinoid System and Neuroinflammation

Microglial cells’ actions significantly contribute to the development of amyloid plaques and neurofibrils through inflammation induction. The role of CB_2_Rs and microglia in AD is intricate yet pivotal for potential therapies. Significant elevation in microglial and astroglial CB_2_R expression was found in a mouse model of AD-related cerebral amyloidosis, highlighting the potential role of CB_2_R as a convenient target for imaging neuroinflammation [43]. Moreover, Tang et al. [44] investigated the relationship between CBRs and AD-related neuroinflammation in SAMP8 mice. Their research revealed that overexpression of microRNA-139 (miR-139), observed in the hippocampus of AD mice, impaired spatial memory, object recognition, fear response, and reactions to pro-inflammatory stimuli by inhibition of intercellular adhesion molecule 1 (ICAM-1) and cluster of differentiation 40 (CD40), and reduction in interleukin-6 (IL-6) and tumor necrosis factor-a (TNF-a). This effect was attributed to the regulation of *CB_2_R* gene expression by miRNAs, indicating the involvement of CB_2_R-mediated neuroinflammatory processes in AD’s neurotoxic effects. Additionally, the role of CB_2_R in regulating NOD-like receptor family pyrin domain containing 3 (NLRP3) mediated neuroinflammation in astrocytes was also determined as proof of the relationship between CB_2_R and neuroinflammation, revealing that the activation of the NLRP3/Caspase-1/IL-1β pathway was enhanced in astrocytic CB_2_R knockdown mice, whereas the deposition of NLRP3 on astrocytes declined following receptor activation [45].

Overexpression of CB_2_R in these cells underscores its crucial role in limiting neuroinflammation [46]. While persistent microglial activation, known as chronic microgliosis, typically worsens neuroinflammation and neuronal damage in AD, activating CB_2_Rs shows neuroprotective effects, as evident from several studies discussed below (Section 3.2 and Section 3.3). Recent research indicates CB_2_R agonists can modulate microglial activity, reducing the release of inflammatory cytokines elevated in AD, thereby shielding neurons from inflammation-induced harm. CB_2_R activation also enhances microglial clearance of Aβ plaques, reducing their neurotoxic effects [46,47]. Thus, this dual role of microglia, where they can be both detrimental through chronic activation and beneficial when appropriately modulated, highlights CB_2_R as a promising therapeutic target, balancing their response to mitigate neuroinflammation and promote neuroprotection in AD. 

Thus, despite the known adverse effects of cannabinoids on various bodily systems, including the central nervous, respiratory, cardiovascular, and skeletal systems, recent research has explored their potential in combating diseases like epilepsy, psychotic episodes, Parkinson’s disease, anxiety disorders, depression, and AD.

### 2.3. Proteomic Evidence for the Involvement of the Endocannabinoid System in Alzheimer’s Disease

Given the ECS dysfunction in AD, proteomic studies are currently being conducted to explore the effects of several cannabinoids on AD, identifying several key pathways and proteins that they may influence. Briefly, cannabinoids have been shown to interact with neuroinflammatory pathways, which play a crucial role in AD progression. They modulate the activation of microglia, reducing the release of proinflammatory cytokines such as IL-1β, TNF, and IL-6, thereby preventing further neuronal damage and Aβ plaque formation. Additionally, cannabinoids affect the nuclear factor kappa-light-chain-enhancer of activated B cells (NF-κB) and MAPK pathways, which are involved in inflammatory response and cellular stress, potentially reducing TAU hyperphosphorylation and Aβ accumulation. Specific receptors like transient receptor potential cation channel subfamily M member 2 (TRPM2) and triggering receptor expressed on myeloid cells 2 (TREM2) are targeted by cannabinoids to mitigate neuroinflammation and promote neuronal survival. Genetic variants such as sialic acid-binding Ig-like lectin 3 (*CD33*), *TREM2*, and complement receptor type 1 (*CR1*) are also implicated as risk factors for neuroinflammation. Advanced proteomic analyses reveal links between these genetic contributors and malfunctioning signaling pathways, including upregulation of factors like TNF-a, transforming growth factor-β (TGF-β), and IL-1α, promoting proinflammatory mechanisms via intracellular signaling and trafficking, synaptic function, and cell metabolism/proliferation [48].

Research has also identified transcription factor EB (TFEB) as a critical player in the protective action of a novel CB_2_R bitopic ligand, FD22a, against Aβ-induced harm in glial cells, implicating pathways involved in autophagy and lysosomal biogenesis regulated by TFEB [49]. Other proteomic studies highlight proteins such as IL-1β, TNF, and cyclooxygenase-2 (COX-2) in the context of neuroinflammation; superoxide dismutase (SOD), glutathione peroxidase (GPx), and catalase (CAT) for oxidative stress response; and brain-derived neurotrophic factor (BDNF), synaptophysin, and postsynaptic density protein 95 (PSD-95) for synaptic plasticity, suggesting cannabinoids’ potential neuroprotective effects [50]. Furthermore, the study by Wang et al. [51] found that deletion of CB_2_R in mice exacerbated Aβ neurotoxicity by downregulating key Aβ degradation enzymes, specifically angiotensin-converting enzyme (ACE) and insulin-degrading enzyme (IDE), increasing Aβ levels and associated neurotoxicity. CB_2_R activation, conversely, decreased Aβ levels and increased ACE and IDE levels, highlighting CB_2_R’s protective role in AD by promoting Aβ degradation.

As science continues to advance, it both addresses existing questions and raises new ones, leading to ongoing research. Despite the rapid pace of scientific inquiry, gaps in knowledge persist, often due to limited study or understanding. These gaps are particularly evident in the search for effective treatments for diseases like AD. Consequently, there is a growing need to explore alternative therapeutic approaches, leveraging the expanding use of medicinal cannabis and deepening understanding of the ECS. The abovementioned association between CBRs and cognitive functions has prompted a shift in scientific focus towards identifying new cannabinoids and optimizing known ones for Alzheimer’s treatment, as relevant studies continue to give hope. Therefore, this review aimed to synthesize a wide range of studies of the last decade, highlighting promising CBR agonist molecules and their mechanisms of action in reversing AD symptoms. Ultimately, the goal was to contribute to the development of innovative therapeutic models and enrich the scientific literature in this field.

## 3. Therapeutic Potential of Cannabinoids in Alzheimer’s Disease

### 3.1. Selective Agonists of Cannabinoid Receptor 1 (CB_1_R)

Several studies have explored the therapeutic potential of CB_1_R agonists in models of AD, focusing on various aspects of memory impairment and neuroprotection. Crunfli et al. [52] investigated the effects of arachidonyl-2’-chloroethylamide (ACEA), a CB_1_R agonist, in neuro-2a neuroblastoma cells and streptozotocin (STZ)-induced AD models. They observed significant cognitive improvement with ACEA treatment, enhancing both short-term and long-term memory. Concurrently, STZ + ACEA-treated rats showed increased insulin receptor levels and antiapoptotic B cell leukemia/lymphoma 2 protein (Bcl-2), alongside decreased protein kinase B (Akt) and extracellular signal-regulated kinase (ERK) activity. ACEA also improved cell viability in STZ-treated cells by more than 30%. On the other hand, Moreira-Silva et al. [53] explored the impact of AEA in the same AD models through intracerebroventricular (i.c.v.) injection. AEA administration mitigated cognitive impairments and prevented cerebroventricular enlargement induced by STZ. Additionally, synaptic transmission components such as synaptophysin and syntaxin, reduced by STZ, were restored post AEA treatment. AEA was also used along other endocannabinoids, namely noladin and O-arachidonylethanolamine (OAE), where their effects on Aβ_42_ accumulation were studied in in vitro models [54]. Significant inhibition of Aβ_42_ accumulation by AEA, noladin, and arachidonic acid were found. Furthermore, these compounds enhanced HT22 cell viability via CB_1_R agonism, albeit with varied efficacy in different cell types.

Additionally, Hosseininia et al. [55] investigated the effects of arachidonylcyclopropylamide (ACPA) and miR-137/let-7a on memory impairment in STZ-induced AD models. ACPA microinjection improved memory across various brain regions, including the hippocampal CA1 region, central amygdala (CeA), and medial prefrontal cortex (mPFC), accompanied by decreased *MAGL* gene expression. Lentiviral miR-137/let-7a administration reversed STZ-induced amnesia by increasing endocannabinoid levels. CB_1_R peptide agonists [(m)RVD-hemopressin (RVD) and (m)VD-hemopressin (VD)], were also investigated in Aβ_1–42_-lesioned and scopolamine-induced AD models [56,57,58]. Both peptides restored memory function dose-dependently in various tests, such as the Novel Object Recognition (NOR) and Object Location Recognition (OLR) tests, and exhibited antioxidant and anti-apoptotic properties, potentially through CB_1_R activation. In elucidating RVD’s mechanism [59], it was found that RVD prevented dysfunction in the BDNF/Tropomyosin receptor kinase B (TrkB)/Akt signaling pathway in HT22 cells treated with scopolamine. This was associated with increased expression of synapsin-1 and PSD-95 proteins, crucial for synaptic plasticity and memory formation. RVD’s efficacy in mitigating Aβ_1–42_-induced TAU protein phosphorylation by inhibiting protein kinase A (PKA) and GSK-3β activity, as well as modulating neuronal growth in SH-SY5Y cells, was also shown [60]. 

Finally, Velikova et al. [61] investigated CB_1_R’s role in memory and learning via agonist (HU-210) and antagonist (SR 141716A) administration in a rat model of olfactory bulbectomy (OBX). HU-210 improved memory, while SR 141716A exacerbated memory deficits, underscoring CB_1_R’s involvement in memory processes.

As evidenced across studies, CB_1_R agonists consistently improved cognitive function in AD models, often through modulation of synaptic proteins, anti-apoptotic pathways, and/or antioxidant effects. However, efficacy varied with different endocannabinoids and peptide agonists, suggesting a need for further comparative studies, especially ones that delve deeper into the mechanisms linking CB_1_R activation to cognitive improvement and neuroprotection.

The main outcomes of the studies involving selective CB_1_R agonists are summarized in Table 1.

### 3.2. Selective Agonists of Cannabinoid Receptor 2 (CB_2_R)

CB_2_R agonists consistently improve cognitive performance across various AD models, while also reducing Aβ deposition, oxidative stress, and inflammatory responses, with specific pathways such as peroxisome proliferator-activated receptor-γ (PPAR-γ), toll-like receptor 4 (TLR4)/NF-κB, and PI3K/Akt, implicated in these effects. However, diverse molecular actions of CB_2_R activation can lead to these effects.

In more detail, Jayant et al. [62] investigated the CB_2_R agonist 1-phenylsatin in mouse models of AD induced by STZ or aluminum chloride (AlCl_3_) + D-galactose (D-Gal), noting that it restored cognitive function and mitigated biochemical (related to oxidative stress) and structural (related to Aβ accumulation) brain lesions. Similarly, the CB_2_R agonist β-caryophyllene (BCP) was found to improve cognitive performance and reduce Aβ deposition in the cerebral cortex and hippocampus, and also various inflammatory markers such as COX-2, IL-1β and TNF-a in APP/PS1 mice, suggesting an anti-inflammatory mechanism via the PPAR-γ pathway [63]. Moreover, Del Cerro et al. [64] demonstrated that the CB_2_R agonist PGN33 decreased the viability of lymphoblasts from late-onset AD patients and mitigated Aβ-induced neuroblastoma cell death, implicating impedance of the Ca^2+^/calmodulin-dependent activation of PI3K/Akt signaling pathway.

Another CB_2_R agonist, JWH-015, was examined for its effects on transgenic APP/PS1 mice, finding enhanced novel object recognition and immunoprotective effects through microglial phenotype conversion from M1 to M2 [65]. This finding aligns with the results of Çakır et al. [66], who observed that JWH-133 reduced escape latency, distance traveled, decline of spatial memory, and inflammatory markers in an okadaic acid (OKA)-induced model of hyperphosphorylated TAU, further suggesting neuroprotective and anti-inflammatory properties of CB_2_R agonists.

Complementarily, the role of CB_2_Rs in regulating glucose uptake in the mouse brain was explored in different models representing brain disorders that involve neurometabolic alterations like AD. This study [67] offered a fresh insight into the beneficial activity of CB_2_R agonism, as AD is characterized by decreased glucose uptake leading to impaired neuronal function, increased Aβ deposition and TAU pathology, and cognitive deficits. Both selective (JWH133, GP1a) and non-selective (WIN55212-2) CB_2_R agonists stimulated glucose uptake in astrocytes and neurons, an effect prevented by the CB_2_R antagonist AM630. This effect was observed across different brain regions of young and middle-aged mice. Additionally, COX-2 inhibition stimulated glucose uptake in middle-aged mice but not in TgAPP-2576 mice, likely due to reduced anandamide levels, suggesting a novel glucoregulatory role for CB_2_Rs.

Finally, a recent study [68] revealed that OX_1_R antagonists could enhance the neuroprotective effects of CB_2_R. It was shown that OX_1_R and CB_2_R form CB_2_-OX_1_-Hets in transfected HEK-293T and microglial APPSw/Ind cells. Co-activation of CB_2_R by JWH-133 and OX_1_R by orexin-A resulted in a “non-additive” decrease in cAMP levels, reversed by CB_2_R antagonism in HEK-293T cells, indicating negative crosstalk and cross-antagonism. On the other hand, OX_1_R antagonism enhanced CB_2_R activation effects. Similar results were observed in AD-model microglial cells, where the expression of the CB_2_R-OX_1_R complex was two-fold higher than in the microglia of control animals.

Even though these results hold promise and the role of CB_2_R agonists in reducing inflammation and oxidative stress and regulating glucose uptake is established, further research is needed to fully elucidate the molecular pathways involved and more comparative studies are required to determine the most effective CB_2_R agonist and dosage for different AD models and stages.

The main outcomes of the studies involving selective CB_2_R agonists are summarized in Table 2.

### 3.3. Agonists of Cannabinoid Receptor 2 (CB_2_R) Associated with Cholinergic Pathways

Several studies have explored the interplay between CB_2_R agonists and cholinergic pathways, emphasizing their impact on memory and cognitive performance. One such study [69] found that combining suboptimal doses of the CB_2_R agonist JWH-133 and the cholinergic receptor agonist nicotine significantly improved cognitive performance in scopolamine-treated mice, while counteracting the cognitive impairment induced by the antagonist.

Additionally, Montanari et al. [70] synthesized 2-arylbenzofuran derivatives, identifying compound **8** as a potent butyrylcholinesterase (BChE) inhibitor and CB_2_R agonist, which demonstrated neuroprotective effects and promoted a shift in microglial cells from an inflammatory (M1) to a neuroprotective (M2) phenotype. Conversely, compound **10** demonstrated robust immunomodulatory activity as an inverse agonist of CB_2_R. Similarly, Spatz et al. [71] synthesized hybrid molecules acting as CB_2_R agonists and BChE antagonists, with compounds **15d** and **21d** showing significant promise. These compounds exhibited immunomodulatory effects by attenuating the inflammatory M1 phenotype in lipopolysaccharide (LPS)-treated microglial cells. Notably, compound **15d** prevented learning impairments in mice challenged with Aβ_25–35_ oligomers, suggesting potential therapeutic utility against AD. This aligns well with the findings of the previous study. Another group [72] also took a similar approach by designing hybrid synthetic analogs of tacrine (AChE inhibitor) and a selective CB_2_R agonist. Compounds **3e**, **4a**, and **8** exhibited neuroprotective effects in a cellular model of neuronal oxidative stress, with compound **8** proving most potent. In AD mouse models, these compounds prevented Aβ_25–35_ infusion-induced memory impairments, demonstrating greater efficacy than the parent molecules and the ability to penetrate the blood–brain barrier.

Therefore, all these studies highlight the potential of combining CB_2_R agonism with cholinergic modulation to improve cognitive performance and mitigate AD-related pathology.

The main outcomes of the studies involving compounds targeting CB_2_R and cholinergic pathways are summarized in Table 3.

### 3.4. Non-Selective Agonists of Cannabinoid Receptors 1 and 2 (CB_1_R and CB_2_R)

In vitro studies with non-selective agonists of CB_1_R and CB_2_R reveal that both Δ^9^-THC and Δ^8^-THC reduce Aβ aggregation and improve cell viability, with Δ^9^-THC also diminishing GSK-3β and TAU levels [73]. Δ^8^-THC was further shown to upregulate proteasome subunits and ubiquitin, suppress the unfolded protein response, reduce Bax, and increase Bcl-2 levels [74]. CBD protects synaptic plasticity through PPAR-γ activation, as observed in slices from the CA1 region of the hippocampus in C57Bl/6 mice using hippocampal long-term potentiation (LTP), a marker of synaptic strength limited by Aβ, to assess the impact. However, the lack of involvement of 5HT_1_A, adenosine (A2A), or CB_1_ receptors was concluded [75]. Additionally, β-amyrin demonstrated anti-inflammatory effects in rat microglial cells treated with LPS/interferon-γ (IFN-γ), where it enhanced cell survival and reduced pro-inflammatory cytokines and COX-2 expression, modulating the gene expression ratio towards an M2 anti-inflammatory state [76]. Similarly, WIN 55,212-2 exhibited significant anti-inflammatory and neuroprotective effects by improving cell viability and reducing pro-inflammatory cytokines, COX-2, and inducible nitric oxide synthase (iNOS) expressions, while increasing PPAR-γ and Cu/Zn SOD expression [77]. WIN 55,212-2 and other cannabinoid agonists also effectively prevented neuronal death by reducing Cx43 hemichannel activity in astroglia and hippocampal pyramidal cells, lowering glutamate and ATP secretion, actions mediated by CB_1_R [21]. Furthermore, CP55-940’s combined use with CB_1_R inverse agonists and anti-Aβ_42_ antibodies in cellular models of familial AD inhibited intracellular APP aggregation and TAU phosphorylation, restored mitochondrial membrane potential, reduced ROS formation, and suppressed apoptosis markers, offering a promising combination strategy for treating familial AD [78]. Finally, the positive effects of the phytocannabinoid cannabinerol (CBNR) on retinoic acid-differentiated SH-SY5Y cells treated with Aβ were recently found, as CBNR partially restored the cell viability, mainly through preventing mitochondrial and endoplasmic reticulum dysfunctions. The transcriptomic analysis revealed 1549 DEGs, mainly related to oxidative phosphorylation (COX6B1, OXA1L, MT-CO2, and MT-CO3), protein folding (HSPA5) and degradation (CUL3, FBXW7, and UBE2D1), and glucose (G6PC3) and lipid (HSD17B7, ERG28, and SCD) metabolism [79].

In the in vivo setting, the studies collectively highlight the potential of various cannabinoids, including CBD, CBDA, and Δ^9^-THCA, and synthetic analogs like NlTyr and WIN55,212-2, in mitigating AD symptoms, improving cognitive functions, and offering neuroprotection and reduction in pathological markers such as Aβ and p-TAU. In more detail, chronic daily administration of a moderate dose of CBD could benefit AD symptoms such as anxiety, cognitive dysfunction, and sensorimotor impairment, while, despite mixed outcomes, CBD administration notably restored spatial learning speed, perseveration, and novel object recognition in female APP_Swe_/PS1ΔE9 (APPxPS1) mice [80]. Nano-chitosan-coated CBD improved learning and memory while increasing the expression of CB_1_R and CB_2_R in the hippocampus, indicating the potential of nano-chitosan to enhance CBD’s effectiveness in cognitive processes [81]. CBD was also found to upregulate genes involved in immune response and autophagy in APP/PS1 mice, suggesting that its therapeutic effects are due to reduced neuroinflammation and enhanced cellular recycling [82]. This aligns with the study by Kim et al. [83], who showed that intrahippocampal injection of CBDA and Δ^9^-THCA in Aβ_1–42_-injected mice improved escape latency, increased the discrimination index, and significantly reduced Aβ polymers and p-TAU levels in the hippocampus, indicating restored cognitive functions and neuroprotective properties. Similarly, the AEA analog, N-linoleyltyrosine (NlTyr), restored motor coordination and improved cognitive and learning abilities, also reducing Aβ_42_ levels in the hippocampus through cannabinoid receptor-mediated autophagy [84]. Finally, WIN55,212-2 enhanced the MWM test outcomes, also reducing malondialdehyde levels, restoring antioxidant molecules glutathione and SOD, and promoting neurogenesis markers nestin and glial fibrillary acidic protein (GFAP) in the hippocampus [85].

An analysis of the clinical studies with cannabinoids shows that Δ^9^-THC [86], CBD [87], and nabilone [88] have all demonstrated potential in improving behavioral symptoms, such as restlessness, irritability, sleep disturbances, and apathy, and cognitive function in dementia and AD patients. CBD and nabilone particularly stood out for their significant reductions in Neuropsychiatric Inventory (NPI) scores and improvements in related assessments, indicating their promise in managing behavioral and psychological symptoms of dementia (BPSD). Δ^9^-THC’s efficacy was less conclusive in two randomized controlled trials [89,90], where several scores such as the NPI and its subscales, the Cohen-Mansfield Agitation Inventory (CMAI), the Quality of Life-Alzheimer’s Disease (QoL-AD), and the Barthel Index, showed no statistically significant differences with the placebo group, but individual case reports [86] and cohort studies with cannabis extracts [32,91] have indicated notable improvements in specific symptoms related to emotional state, behavior, and aggression, and in cognitive functions, suggesting potential benefits in personalized treatment regimens. Additionally, oral Δ^9^-THC exhibited partially good effects on balance and gait in dementia patients with behavioral symptoms, indicating some improvements, such as improved stride length and trunk sway during preferred speed walking, but also increased sway under certain conditions [92]. 

In terms of safety and tolerability, all studies reported good tolerability of cannabinoid treatments, with no severe adverse effects documented. This highlights the safety profile of these compounds in the studied dosages and supports their feasibility for long-term use, though comprehensive safety evaluations in larger populations are essential. The consistency in the lack of significant adverse effects across different cannabinoid treatments reinforces their potential for broader clinical application, pending further validation from larger and longer-term studies. These trials should aim to establish standardized dosing regimens and identify patient subgroups that may benefit most from cannabinoid treatments, as the variability in responses to these treatments underscores the need for personalized approaches. Identifying biomarkers or patient characteristics that predict positive responses can enhance the clinical utility of cannabinoids in dementia and AD.

The main outcomes of the studies involving non-selective agonists of CB_1_R and CB_2_R are summarized in Table 4.

### 3.5. Non-Selective Agonists of Cannabinoid Receptors 1 and 2 (CB_1_R and CB_2_R) Related to Cholinergic Pathways

Nuñez-Borque et al. [93] conducted a study to examine the effects of two CBR agonists, NP137 and NP148, in both immortalized lymphocytes from patients with delayed-onset AD and TgAPP mice. These agonists demonstrated inhibitory effects on β-secretase-1 (BACE-1) and BACE-1/BChE. Initially, the study revealed a significant attenuation of Aβ-induced cell death in neural cortical cells following pretreatment with NP137 or NP148. Moreover, through the MWM test, long-term administration of NP137 to TgAPP mice effectively restored their cognitive functions, evidenced by a reduction in escape latency comparable to that of the control group. Additionally, the addition of NP137 was found to attenuate the increased proliferative activity of AD cells and normalize ERK1/2 phosphorylation and p21 content in AD lymphoblasts.

### 3.6. Molecules that Act through Pathways Related to the Endocannabinoid System

In studies related to receptors associated with the ECS apart from CB_1_R and CB_2_R, the researchers investigated the potential restorative effects of activating cannabinoid receptors to counteract Aβ-induced impairments. Balleza-Tapia et al. [94] focused on the ionotropic cannabinoid receptor Trpv-1, part of the ECS [95], using capsaicin to prevent neuronal degeneration, reverse action potential desynchronization in CA3 pyramidal cells, and restore the balance between excitatory and inhibitory potentials. This demonstrates that Trpv-1 activation can significantly mitigate Aβ-induced impairments in hippocampal cells.

Similarly, Xiang et al. [96] explored the impact of GPR-55 activation, another ECS receptor [97], using the agonist O-1602. Their studies showed that O-1602 mitigated cognitive impairment in Aβ_1–42_-induced neurotoxicity by reducing soluble Aβ_1–42_ levels in the hippocampus and frontal cortex, reversing GPR-55 downregulation, and decreasing levels of Ras homolog family member A (RhoA) and Rho-associated coiled-coil-containing protein kinase 2 (ROCK2) proteins. Further research by the same group [98] revealed that O-1602 also decreased BACE1 activity, oxidative stress markers, and pro-inflammatory cytokines while improving synaptic plasticity through the upregulation of PSD-95 protein and reducing microglial activation in a model of STZ-induced neurotoxicity.

Complementary findings by Wang et al. [99] used the same agonist, O-1602, in LPS-challenged mice to explore its impact on cognitive impairment. They observed that O-1602 attenuated the expression of NF-κB p65, Bax protein, and caspase-3 activity, while increasing Bcl-2 expression and anti-inflammatory cytokines. Additionally, O-1602 significantly reduced hippocampal cell apoptosis, evidenced by fewer TUNEL-positive cells.

Thus, both approaches targeting Trpv-1 and GPR-55 demonstrated significant reductions in neuronal degeneration and cognitive impairments, although through different molecular pathways. The role of Trpv-1 and GPR-55 activation in reducing Aβ-induced impairments and cognitive deficits is established. However, the specific downstream signaling pathways and interactions with other receptors need further exploration.

The main outcomes of the studies involving non-selective agonists of CB_1_R and CB_2_R associated with cholinergic pathways and molecules that act through pathways related to the ECS are summarized in Table 5.

### 3.7. Combination Studies of Agonists of Different Classes

In a number of studies, researchers combine various cannabinoid agents of different classes to evaluate their potential in ameliorating conditions associated with AD through different mechanisms. For example, Elmazoglu et al. [100] focused on a range of cannabinoid agents in a primary rat hippocampal neuron model of toxic hyperglycemia and Aβ_1–42_ treatment. The study aimed to enhance cell viability by activating nuclear factor erythroid 2-related factor 2 (Nrf2) and reducing oxidative stress and inflammation. Among the tested agents, URB597 (a FAAH inhibitor) emerged as the most effective in promoting cell survival and suppressing ROS formation. Synthetic cannabinoids WIN55,212-2 and CP55-940, followed by endocannabinoids 2-AG and AEA, also exhibited efficacy in enhancing cell survival and limiting Aβ aggregation. Furthermore, all tested agents increased antioxidant enzymes, including SOD, CAT, GPx, and glutaredoxin (GRx), along with Nrf2, to mitigate inflammation.

Furthermore, the neuroprotective potential of 11 non-psychoactive cannabinoids was investigated using a preclinical drug screening platform for AD [101]. The researchers conducted various assays in HT22 or MC65 cells after inducing C99 production, representing proteotoxicity, loss of trophic factors, and oxidative stress. Additionally, cannabinoids were examined for their ability to reduce accumulated Aβ. Notably, Δ^9^-THC and Δ^8^-THC demonstrated efficacy in preventing Aβ toxicity. Cannabinoids were also assessed for their capacity to suppress the pro-inflammatory response of microglial cells to LPS, with only CBD, dimethyl cannabidiol, cannabigerolic acid, and Δ^9^-THC exhibiting EC50 values <10 µM. Importantly, this study revealed that the neuroprotection offered by the tested cannabinoids is independent of CB_1_R and CB_2_R activation, as none of the cells in the study expressed these receptors.

Finally, the in vivo effects of prolonged oral administration of synthetic cannabinoids WIN55,212-2 and JWH-133 in TgAPP mice were examined in another study [102], focusing on vascular function alterations within the AD brain. Both agonists normalized the elevated levels of collagen IV-positive vessels in the frontal cortex, reducing collagen IV vascular density. While the cannabinoids’ dilator effect was limited in the aortic valve of TgAPP mice compared to controls, their administration effectively prevented Aβ-induced desensitization of the vasodilatory action of ACh. In a similar long-term study on the effects of oral JWH-133 or Cannabixir^®^ Medium Flos with or without donepezil in APP/PS1 mice, the treatments ameliorated cognitive decline and anxiety-like behavior, also reducing the size and amount of Aβ plaques, cerebral glucose metabolism, and expression of mTOR and CB_2_R, enlarging astrocytes and upregulating M1 AChR expression [103]. 

Therefore, the importance of antioxidant enzymes and reduced ROS formation in mitigating AD-related damage was highlighted and all studies, suggesting that enhancing antioxidant defenses and reducing oxidative stress are common mechanisms through which cannabinoids confer neuroprotection. Navarro-Dorado et al.’s [102] findings align with the other studies in demonstrating the neuroprotective effects of cannabinoids in an in vivo setting. However, their focus on vascular function adds another dimension to understanding how cannabinoids can mitigate AD symptoms. The normalization of collagen IV vascular density and prevention of Aβ-induced desensitization of ACh’s vasodilatory action indicate that cannabinoids also contribute to maintaining vascular health, which is crucial for cognitive function.

The main outcomes of the combination studies with agonists from different classes are summarized in Table 6.

## 4. Discussion

Alzheimer’s disease continues to pose a significant challenge due to its rising prevalence and the limited effectiveness of current therapeutics. The exploration of cannabinoids as neuroprotective and therapeutic agents offers a promising avenue for addressing the unmet needs in AD treatment. This review article aimed to synthesize the latest research on cannabinoids in AD, providing a comprehensive overview that underscores their potential benefits. Complementary to the existing literature, we believe that this review is crucial for consolidating recent findings and guiding future research directions in this evolving field.

The analysis focused on the therapeutic potential of various cannabinoids in AD that target either CB_1_R and CB_2_R or Trpv-1 and GPR-55. It was collectively shown that CB_1_R selective agonists, such as ACEA, AEA, Noladin, OAE, RVD, VD, and HU-210, demonstrated positive effects on cell viability and memory function. RVD and VD notably increased antioxidant enzymes, reducing oxidative stress via the BDNF/TrkB/Akt pathway. Co-administration of ACPA and miRNA-137/-let-7a lentiviral particles enhanced memory function by increasing endocannabinoids through MAGL downregulation. Studies on CB_2_R selective agonists also highlighted their role in memory recovery, anti-inflammatory effects, and immunomodulatory properties, possibly via the TLR4/NF-κB p65 pathway. Furthermore, synthetic CB_2_R agonists combined with AChE/BChE inhibition showed significant neuroprotection without psychotropic effects. Non-selective CB_1_R/CB_2_R agonists on the other hand, including Δ^9^-THC, CBD, and various *Cannabis sativa* extracts, revealed mixed results for Δ^9^-THC but consistent positive outcomes for CBD in reversing AD traits through PPAR-γ receptor activation. Clinical trials on nabilone showed its effectiveness in improving cognitive functions and managing neuropsychiatric symptoms, despite potential sedation. Synthetic cannabinoids like WIN-55,212-2 and CP55-940 demonstrated neuroprotective effects and reduced inflammation, supporting their therapeutic potential. Additionally, compounds targeting receptors indirectly involved in the ECS, such as Trpv-1 and GPR-55, exhibited neuroprotective and anti-inflammatory properties, indicating broader intervention targets for AD treatment. Therefore, there is a clear need to advance these studies further and in more clinical trials in order to come up with more effective and targeted cannabinoid therapeutics. 

**Figure 1:**
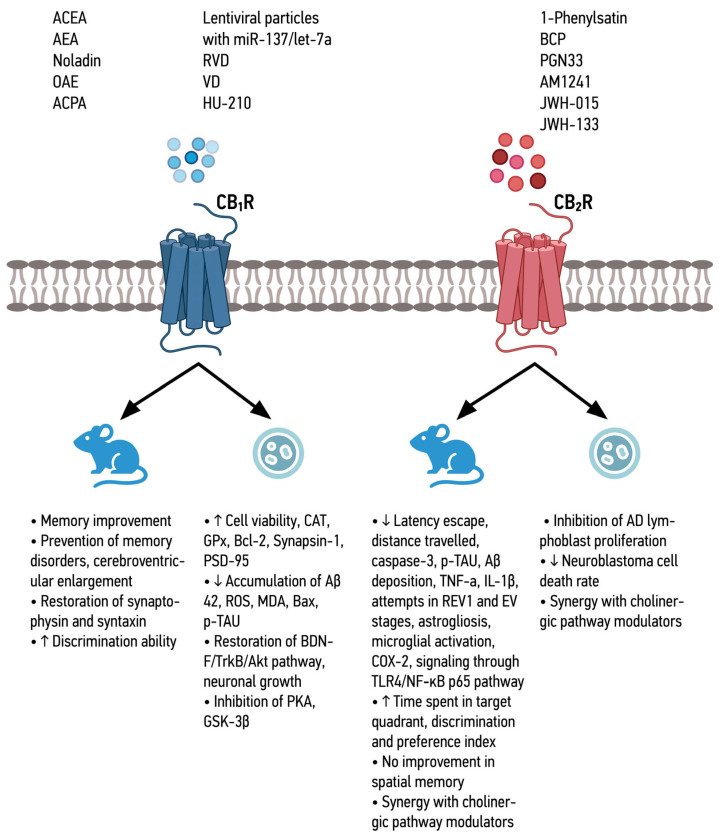
Summary of the effects of CB_1_R and CB_2_R agonists in in vitro and in vivo models.

**Figure 2:**
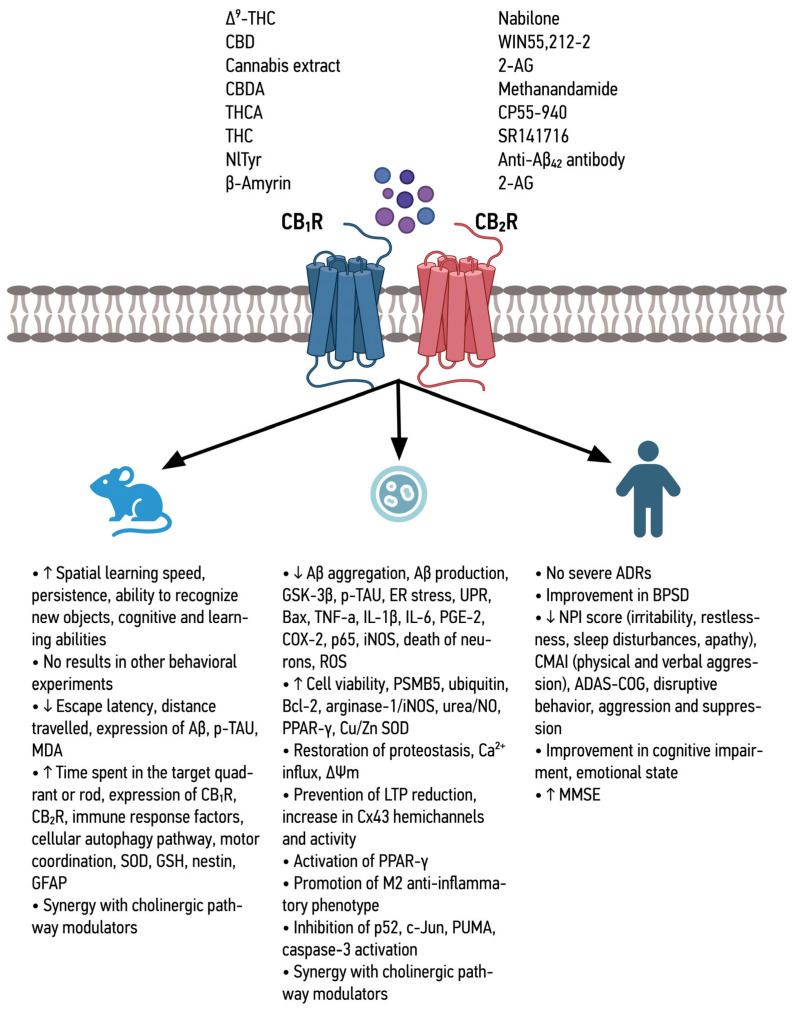
Summary of the effects of non-selective CB_1_R and CB_2_R agonists in in vitro and in vivo models and in patients with Alzheimer’s disease.

**Figure 3:**
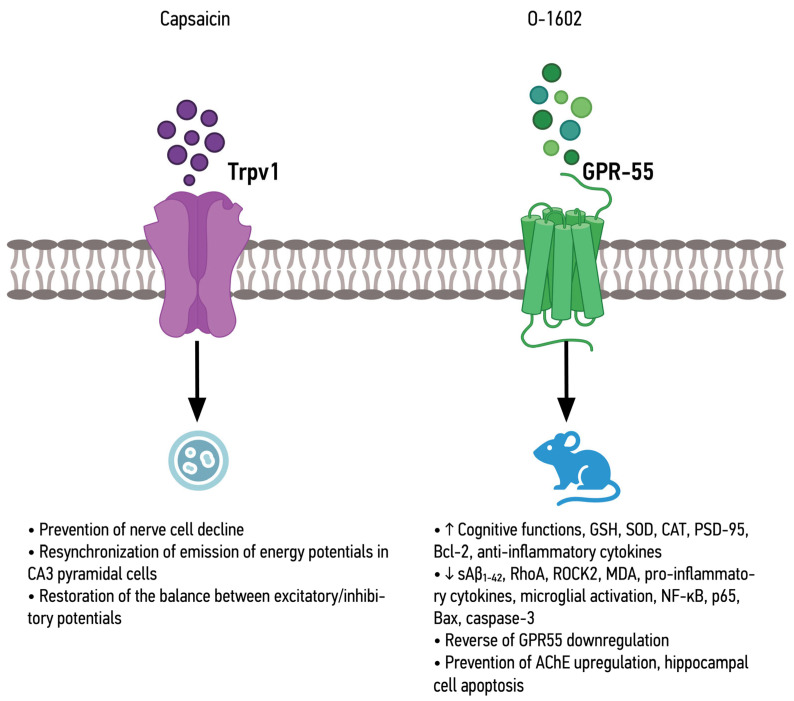
Summary of the effects of TRPV-1 and GPR-55 agonists in in vitro and in vivo models.

The combined effects of the different CBR agonists (Figure 1 and Figure 2), as well as Trpv-1 and GPR-55 agonists (Figure 3), are shown below. 

As is evident from the above studies, different research groups chose to work with different experimental models, which might pose a limitation in the interpretation and clinical translation of the observed results, not only for cannabinoids but also for any other treatment under development. In these regards, and while experimental models of AD are invaluable for understanding the disease and testing potential therapies, each model has its drawbacks that can influence the observed results in a study. For instance, the STZ injection model mimics sporadic AD in rats through insulin signaling impairment but lacks amyloid and TAU pathology, which are hallmarks of human AD. Similarly, the rapid induction of amyloid pathology in mice infected with Aβ_1–42_ and Aβ_25–35_, while useful for studying amyloid-related mechanisms, does not replicate the slow, progressive nature of the disease. Scopolamine-induced AD in rats, focusing on temporary cholinergic deficits, and OBX rats, emphasizing neuroinflammation, offers valuable insights but may not fully encompass the multifaceted pathology of AD. Furthermore, transgenic models like APP/PS1 and TgAPP mice provide important information on familial AD and amyloid pathology but may not fully translate to the more common sporadic, late-onset AD due to their overexpression of mutant proteins and the early-onset nature. The AlCl_3_ + D-Gal-induced AD model and okadaic acid-induced AD in rats introduce non-specific toxicity and acute TAU pathology, respectively, which do not perfectly mirror human AD’s chronic progression. These limitations suggest that the therapeutic effects observed in these models might not fully capture the complexity of AD, as the pathogenesis and progression in humans involve multiple interacting pathways over a prolonged period [104,105,106,107,108].

Despite these drawbacks, the combined use of these models is crucial for a comprehensive understanding of AD and the potential therapeutic effects of cannabinoids. Each model highlights different aspects of the disease, such as amyloid aggregation, TAU phosphorylation, neuroinflammation, oxidative stress, and cognitive deficits, allowing for a broad evaluation of cannabinoids. Researchers must consider these limitations and interpret results within the context of each model’s specific characteristics, ensuring a cautious and well-rounded approach when translating findings to human AD.

Given cannabinoids’ potential in AD, researchers have also started to explore them in other less frequent neurodegenerative and related diseases that share common traits with AD. Such a disorder is progressive supranuclear palsy (PSP), which also induces cognitive decline, changes in behavior, mood, personality, and difficulties in performing everyday tasks. In a relevant case report of a 71-year-old woman with PSP who experienced severe motor and language impairments, cannabis treatment led to significant improvements in balance, gait, and language abilities, along with an overall better quality of life with reduced muscle stiffness. The proposed mechanisms behind these improvements include the anti-inflammatory and neuroprotective effects of cannabinoids, as well as their muscle relaxant properties, which may help reduce neuroinflammation and muscle rigidity [109]. Similarly, a case series on the effects of cannabinoids on patients with frontotemporal dementia, specifically those with the behavioral variant, showed notable improvements in their behavioral symptoms such as disinhibition, obsessive/compulsive behaviors, anxiety, insomnia, and pain [110]. Thus, the field of cannabinoid research is still in its infancy and widening their applications in other neurodegenerative disorders will be fundamental in advancing their therapeutic potential.

Finally, in comparison to Cannabis-based treatments that have consistently shown promise in reducing AD’s major pathophysiological characteristics without significant psychoactive side effects, one must consider the other novel therapeutics that have emerged recently. In this regard, 5-HT6 receptor antagonists, such as idalopirdine, target serotonin receptors and have been investigated for their potential to enhance cognitive function by increasing ACh release. However, clinical trials have shown mixed results, with some studies indicating minimal-to-no cognitive improvement in patients [111]. Similarly, alpha-2 adrenergic agonists aim to improve cognitive function by modulating norepinephrine release, which can enhance attention and working memory. Clonidine and guanfacine are examples of this class, but their effectiveness in Alzheimer’s patients has been limited and they present side effects such as hypotension and sedation [112,113,114].

## 5. Conclusions and Future Directions

As understood from the above, cannabinoids exhibit efficacy in reversing several of the manifestations of AD. The number of included studies, both in laboratory settings and clinical trials, provides a rather solid foundation for drawing reliable conclusions. The involvement of several cellular pathways as well as cannabinoid receptors in their mechanism of action holds promise for AD treatment, requiring further investigation. This investigation should also consider the synergistic effect between cannabinoids and other therapeutic methods, such as anti-Aβ_42_ antibodies and anticholinesterase agents, which have shown promising results. Given the current challenge of treating AD, the therapeutic potential of cannabinoids presents a new focus for research in this field.

However, several key considerations must be addressed to advance the translation of preclinical findings into clinically meaningful outcomes. The following future directions outline crucial areas of focus for researchers, clinicians, and regulatory agencies:

1. Even though our review article thoroughly presented the key findings of each included study aiming at elucidating cannabinoids’ mechanisms of action in AD, further exploration of the molecular mechanisms underlying these beneficial effects is imperative. Understanding specific pathways involved in neuroprotection, neuroinflammation modulation, and amyloid plaque reduction will facilitate the development of targeted therapeutic strategies.
2. Given their diverse physicochemical characteristics, standardization of cannabinoid formulations is essential to ensure consistency in dosing and efficacy across studies and clinical trials. Addressing variability in cannabinoid composition, purity, and delivery methods is paramount for reliable and reproducible results.
3. Based on divergent observations in the limited clinical trials and case reports discussed, the optimization of dosage and treatment regimens based on preclinical and early clinical data is necessary. Conducting dose-ranging studies will help identify the most effective and safe doses for AD patients, considering individual variability and disease progression. Apart from that, well-designed, placebo-controlled clinical trials with sufficient statistical power are urgently needed to evaluate the efficacy and safety of cannabinoid-based interventions in AD. Consideration of patient selection criteria, outcome measures, trial duration, and follow-up assessments is essential for robust clinical evidence.
4. Rigorous safety assessments are crucial to address concerns regarding potential adverse effects and long-term consequences of cannabinoid use in AD. Comprehensive evaluation of cognitive, psychiatric, and addictive risks is essential for patient safety. Studies have shown that long-term cannabis use is associated with impairments in learning, memory, and executive functions, with regular users exhibiting declines in IQ and cognitive processing speed over time. Chronic exposure to cannabinoids can lead to structural changes in brain regions critical for memory, such as the hippocampus. The Coronary Artery Risk Development in Young Adults (CARDIA) study found that regular cannabis users exhibit declines in cognitive processing speed and verbal memory over time [115]. Another study showed that chronic cannabis exposure can lead to structural changes in the hippocampus, contributing to memory deficits. Heavy cannabis use is associated with hippocampal thickness abnormalities, particularly in older adults, affecting memory formation and retention [116]. Furthermore, cannabis use can impair verbal memory and executive function, disrupting neural processes related to memory encoding and retrieval. These findings highlight the potential risks for cognitive health with sustained cannabis consumption, indicating that long-term use may contribute to cognitive decline, particularly in memory-critical brain areas [117]. Therefore, safety assessment cannot be overlooked and a careful balance between benefit and risk should be maintained.
5. As discussed throughout this article, cannabinoids exhibit synergistic effects with other therapeutic agents that affect cholinergic neurotransmission by inhibiting AChE or BChE or by inhibiting BACE-1 or FAAH, among others. These combinations may enhance treatment outcomes in AD, thereby reducing the required doses of both agents. Additionally, cannabinoids should be explored along with NMDA receptor antagonists in preclinical and clinical settings.
6. Last but not least, addressing misconceptions and stigmas surrounding cannabinoid use is crucial for fostering acceptance and support for cannabinoid-based therapies in AD. Education, public awareness campaigns, and destigmatization efforts are essential to garnering broader societal acceptance. In these regards, we should not overlook that the legal landscape surrounding the use of cannabis varies significantly across the globe. In countries like Canada and Australia, strict regulations are enforced by Health Canada and the Therapeutic Goods Administration (TGA), respectively. These regulations dictate that cannabis-based treatments must undergo rigorous testing and approval processes before being authorized for medical use. Health Canada, for instance, requires cannabis products to be approved under the Cannabis Act and meet specific quality standards. In contrast, some countries such as the Netherlands and Uruguay have adopted more open-minded approaches, allowing cannabis for medicinal purposes under certain conditions. These nations have established frameworks that permit medical cannabis use, often with physician oversight and specific regulations governing cultivation, distribution, and patient access. However, even in countries with more liberal policies, legal frameworks and regulations continue to evolve as research on cannabis and AD progresses, balancing therapeutic potential with concerns about safety, efficacy, and abuse potential.

In conclusion, while the beneficial effects of cannabinoids in AD are promising, careful consideration of future directions is imperative to ensure responsible and evidence-based advancement. By addressing mechanistic insights, standardization, safety concerns, clinical trial design, ethical considerations, translational challenges, and public perception, the field can progress towards realizing the therapeutic potential of cannabinoids for AD patients. 

## Figures and Tables

**Table 1:** Main results from the studies with selective CB_1_R agonists.

Reference	Cannabinoid	Dosage	Major Results		Model Used
Crunfli et al., 2019 [52]	ACEA	3 mg/kg i.p.	↑ Short-term memory ↑ Long-term memory ↑ Cell viability		In vitro and in vivo experiments in AD models with STZ
Moreira-Silva et al., 2018 [53]	AEA	100 ng i.c.v.	Repair of new object recognition impairments and prevention of non-associative emotional memory disorders (NOR, EPM tests) Prevention of cerebroventricular enlargement Restoration of synaptophysin and syntaxin levels		In vivo experiments in Wistar rats with STZ injection
Khavandi, Rao and Beazely, 2023 [54]	AΕA	10 μΜ	↑ Cell viability ↓ Accumulation of Aβ_42_		In vitro experiments in mouse hippocampal HT22 cells and hamster ovary CHO cells expressing human CB_1_R
	Noladin	10 μΜ	↑ Cell viability ↓ Accumulation of Aβ_42_		
	OAΕ	1 μM 10 μΜ	↑ Cell viability		
Hosseininia et al., 2023 [55]	ACPA	10 ng/0.5 μL (corticolimbic microinjection)	↑ Step-through latency by ACPA, independent of injection region (CA1, CeA, mPFC) ↓ *MAGL* expression by miR-137/let-7a Elimination of amnesiac effect of STZ		In vivo experiments in Wistar rats after i.c.v. STZ
	Lentiviral particles with miR-137 or miR-let-7a	0.5 μL/rat e.o.d.			
Zhang et al., 2016 [56]	RVD and VD	1 nmol 2.5 nmol 5 nmol, i.c.v./i.p.	↑ Discrimination index (NOR, OLR)		In vivo experiments in mice infected with Aβ_1-42_
Zhang et al., 2021 [57]			Dose-dependent restoration of memory function (NOR, OLR)		In vivo experiments in mice after i.p. scopolamine
Zhang et al., 2020a [58]	VD		↑ Cell viability ↓ ROS, MDA ↑ CAT, GPx ↓ Bax, ↑ Bcl-2		In vitro experiments on hippocampal neurons from mice infected with Aβ_1-42_
Zhang et al., 2023 [59]	RVD		↑ Cell viability ↓ ROS, MDA ↑ CAT, GPx ↓ Bax, ↑ Bcl-2 Restoration of BDNF/TrkB/Akt pathway ↑ Synapsin-1, PSD-95		In vitro experiments in HT22 cells treated with scopolamine
Zhang et al., 2020b [60]			↑ Cell viability ↓ Phosphorylation of TAU Inhibition of PKA, GSK-3β Restoration of neuronal growth		In vitro experiments in SH-SY5Y cells infected with Aβ_1-42_
Velikova, Doncheva and Tashev, 2020 [61]	HU-210	5 μg/day i.c.v.	Improvement in memory	Direct correlation of CB_1_R with memory function (active and PA tests)	In vivo experiments in OBX rats
	SR 141716A (CB_1_R Antagonist)	3 μg/day i.c.v.	Deterioration of memory		

Abbreviations: Aβ: amyloid beta; ACEA: arachidonyl-2′-chloroethylamide; ACPA: arachidonylcyclopropylamide; AD: Alzheimer’s disease; AEA: anandamide; Akt: protein kinase B; Bax: Bcl-2-associated X protein; Bcl-2: B cell leukemia/lymphoma 2 protein; BDNF: brain-derived neurotrophic factor; CAT: catalase; CB_1_R: cannabinoid receptor 1; CeA: central amygdala; e.o.d.: every other day; EPM: elevated plus maze; GPx: glutathione peroxidase; GSK-3β: glycogen synthase kinase-3 beta; i.c.v.: intracerebroventricular; i.p.: intraperitoneally; MAGL: monoglycerol lipase; MDA: malondialdehyde; miR: microRNA; mPFC: medial prefrontal cortex; NOR: novel object recognition; OAE: O-arachidonylethanolamine; OBX: olfactory bulbectomy; OLR: object location recognition; PA: passive avoidance; PKA: protein kinase A; PSD-95: postsynaptic density protein 95; ROS: reactive oxygen species; RVD: (m)RVD-hemopressin; STZ: streptozotocin; TrkB: tropomyosin receptor kinase B; VD: (m)VD-hemopressin.

**Table 2:** Main results from the studies with selective CB_2_R agonists.

Reference	Cannabinoid	Dosage	Major Results		Model Used
Jayant et al., 2016 [62]	1-Phenylsatin	20 mg/kg p.o.	MWM test	↓ Latency escape ↑ Time spent in the target quadrant	In vivo experiments in rats exposed to STZ or AlCl_3_ + D-Gal
			Attentional set shifting test	↓ Attempts in REV1, EV stages	
Cheng, Dong and Liu, 2014 [63]	BCP	48 mg/kg p.o.	↓ Escape latency, distance traveled (MWM) Dose-dependent ↓ in Aβ deposition in cerebral cortex, hippocampus ↓ Astrogliosis, microglial activation, COX-2 ↓ TNF-a, IL-1β		In vivo experiments in APP/PS1 mice
del Cerro et al., 2018 [64]	PGN33	2.5 nM 5 nM 7.5 nM 10 nM	Dose-dependent inhibition of uncontrolled proliferation of AD lymphoblasts ↓ Neuroblastoma cell death rate		In vitro experiments in lymphoblasts isolated from AD patients and in Aβ-treated SH-SY5Y cells
Li et al., 2019 [65]	JWH-015	0.5 mg/kg i.p.	Improvement in recognition of new objects, no improvement in spatial memory (NOR, MWM) Inhibition of cortical microglia activation, conversion from M1 to M2 phenotype		In vivo experiments in transgenic APP/PS1 mice
Çakır et al., 2019 [66]	JWH-133	0.2 mg/kg i.p.	↓ Escape latency, distance traveled (MWM) ↓ Caspase-3, p-TAU, Aβ, TNF-a, IL-1β		In vivo experiments in OKA-treated rats with hyperphosphorylated TAU
Köfalvi et al., 2016 [67]	JWH-133	30 nM–1 μΜ	↑ Glucose uptake in hippocampal astrocytes and neurons in vitro and hippocampal slices of young and middle-aged mice ex vivo by CB_2_R agonists, blocked by AM630 ↑ Glucose uptake in 12-month-old wild-type mice by selective agonists and COX-2 inhibitor, blocked by AM630 ↑ Glucose uptake in TgAPP 2567 mice only by CB_2_R agonists ↑ Brain glucose uptake in middle-aged mice by JWH133		In vitro experiments in primary cortical astrocytes and neurons, and acute hippocampal slices and in vivo experiments in young adult male C57Bl/6j and CD-1 mice, middle-aged C57Bl/6j mice, TgAPP-2576 mice
	GP1a (non-selective CB_2_R agonist)	100 nM			
	AM630 (CB_2_R antagonist)	1 μΜ			
Raïch et al., 2022 [68]	JWH-133	100 nM	Formation of CB_2_-OX_1_-heteromers Non-additive ↓ in cAMP levels by co-activation of CB_2_R and OX_1_R Potentiation of CB_2_R activation by OX_1_R antagonism ↑ Expression of CB_2_-OX_1_-heteromers in AD-model microglia		In vitro experiments in HEK-293T cells and microglial APPSw/Ind cells and in vivo experiments in APPSw/Ind transgenic mice

Abbreviations: Aβ: amyloid beta; AD: Alzheimer’s disease; AlCl_3_: aluminum chloride; BCP: β-caryophyllene; COX-2: cyclooxygenase-2; D-Gal: D-galactosidase; EV: extra-dimensional; IL-1β: interleukin-1β; i.p.: intraperitoneally; MWM: Morris water maze; NF-κB: nuclear factor-κB; NOR: novel object recognition; OKA: okadaic acid; p.o.: per os; p-TAU: phosphorylated TAU; REV1: reversal 1; TLR4: toll-like receptor 4; TNF-a: tumor necrosis factor-a.

**Table 3:** Main results from the studies with CB_2_R and cholinergic pathway modulators.

Reference	Cannabinoid	Dosage	Major Results	Model Used
Marta, Agnieszka and Grazyna, 2022 [69]	JWH-133	0.25 mg/kg i.p.	↑ Cognitive ability after co-administration of suboptimal doses of the two agonists (PA tests) Co-administration of a cholinergic antagonist reversed the results	In vivo experiments in scopolamine-treated Swiss mice
	Nicotine (Cholinergic agonist)	0.05 mg/kg s.c.		
Montanari et al., 2021 [70]	Compound **8** (CB_2_R agonist, BChE inhibitor)	5 μΜ	Amelioration of cholinergic impairment Neuroprotection against Aβ_1–42_ oligomers Conversion of microglial cells from M1 to M2 phenotype	In vitro experiments in SH-SY5Y cells treated with Aβ_1–42_
	Compound **10** (CB_2_R inverse agonist)	5 μΜ	Strong immunomodulatory effect	
Spatz et al., 2023 [71]	Compound **15d** (CB_2_R agonist, BChE inhibitor)	0.3–3 mg/kg/day i.p.	Immunomodulatory effect, ↓ M1 phenotype of microglial cells IC_50_ BChE = 0.62 µM, EC_50_ CB_2_R = 244 nM Prevention of learning disorders (spontaneous alternation Υ maze test, PA test)	In vitro experiments in LPS-treated N9 microglial cells and in vivo experiments in mice challenged with Aβ_25–35_ oligomers
Scheiner et al., 2019 [72]	Compound **3e** (CB_2_R agonist, AChE inhibitor)	0.3 mg/kg i.p., o.d.	Neuroprotective action in a cellular model of oxidative stress Compound **8** maximum efficacy, compound **4a** toxic at doses > 5 μM Prevention of alterations in memory and learning by injection of Aβ2_5–35_ (Υ maze test, PA test, behavioral tests)	In vitro experiments in cellular model of neuronal oxidative stress in N9 microglial cells and in vivo experiments in Aβ_25–35_-injected AD mice
	Compound **4a** (CB_2_R agonist, AChE inhibitor)	1 mg/kg i.p., o.d.		
	Compound **8** (CB_2_R agonist, AChE inhibitor)	0.3 mg/kg i.p., o.d.		

Abbreviations: Aβ: amyloid beta; AChE: acetylcholinesterase; AD: Alzheimer’s disease; BChE: butyrylcholinesterase; CB_2_R: cannabinoid receptor 2; i.p.: intraperitoneally; LPS: lipopolysaccharide; o.d.: once daily; PA: passive avoidance; s.c.: subcutaneously.

**Table 4:** Main results from the studies with non-selective CB_1_R and CB_2_R agonists.

Reference	Cannabinoid	Dosage	Major Results	Model Used
In vitro studies				
Cao et al., 2014 [73]	Δ^9^-THC	0.25 nM–2.5 μΜ every 6 or 24 or 48h	↓ Aβ_40_ levels time- and dose-dependently, inhibition of Aβ_40_ aggregation ↓ Aβ protein production after administration every 24h ↓ GSK-3β, TAU phosphorylation, dose-dependently	In vitro experiments in N2a/AβPPswe cells
Gugliandolo et al., 2023 [74]	Δ^8^-THC	20 μΜ	↑ Cell viability ↓ ER stress Restoration of proteostasis ↑ Expression of PSMB5, ubiquitin, Bcl-2 ↓ UPR, expression of Bax	In vitro experiments in SH-SY5Y cells treated with Aβ_1–42_
Hughes and Herron, 2019 [75]	CBD	10 μΜ	Prevention of LTP reduction Therapy due to activation of PPAR-γ, no involvement of CB_1_R	In vitro experiments in sections from CA1 hippocampus region of Aβ-treated C57Bl/6 mice
Askari et al., 2018 [76]	β-Amyrin	4–16 μΜ	↑ Cell viability ↓ Levels and expression of TNF-a, IL-1β, IL-6, PGE-2, and COX-2 ↑ Arginase-1/iNOS, urea/NO ratios Promotion of M2 anti-inflammatory phenotype	In vitro experiments in LPS/IFN-γ-treated rat microglial cells
Aguirre-Rueda et al., 2015 [77]	WIN55,212-2	10 μΜ	↑ Cell viability, ↓ pro-inflammatory cytokines (IL-1β, TNF-a) ↓ Expression of p65, COX-2, iNOS ↑ Expression of PPAR-γ, Cu/Zn SOD	In vitro experiments in rat cortical astrocytes treated with Aβ
Gajardo-Gómez et al., 2017 [21]	WIN55,212-2	5 μΜ	Prevention of increase in Cx43 hemichannels ↓ Cx43 activity in hippocampal astroglial and pyramidal cells, compared to Aβ group ↓ Death rates of pyramidal neurons compared to the Aβ group Implication of CB_1_R in the observed results	In vitro experiments in cells from rat hippocampal slices treated with Aβ_25–35_
	2-AG			
	Methanandamide			
Soto-Mercado et al., 2021 [78]	CP55-940	1 μΜ	Inhibition of sAβPPβf aggregation and TAU phosphorylation Restoration of ΔΨm to normal levels ↓ ROS Inhibition of p52, c-Jun, PUMA, caspase-3 activation Restoration of Ca^2+^ influx function only after co-administration of anti-Aβ_42_ antibody	In vitro experiments in PSEN1 E280A cells (familial AD model)
	SR141716 (CB_1_R inverse agonist)			
	Anti-Aβ_42_ antibody			
	2-AG			
	CP55-940			
	WIN55,212-2			
	URB597 (FAAH inhibitor)			
Chiricosta et al., 2024 [79]	Cannabinerol	20 μΜ	↑ Cell viability Restoration of mitochondrial function by regulating genes involved in oxidative phosphorylation Endoplasmic reticulum function improvement by regulating genes related to protein folding and degradation Metabolic regulation by regulating genes related to glucose and lipid metabolism	In vitro experiments in SH-SY5Y cells treated with Aβ
In vivo studies				
Coles et al., 2020 [80]	CBD	5 mg/kg i.p.	↑ Spatial learning speed, persistence, ability to recognize new objects Unsuccessful results in a variety of other behavioral experiments	In vivo experiments in female APP/PS1 mice
Amini and Abdolmaleki, 2022 [81]	CBD with nano-chitosan coating	120 mg/kg p.o.	↓ Escape latency, distance travelled, ↑ time spent in the target quadrant (MWM test) ↑ Expression of CB_1_R, CB_2_R in the hippocampus	In vivo experiments in Aβ_1–42_-treated rat AD model
Hao and Feng, 2021 [82]	CBD	5 mg/kg/day i.p.	Upregulation of immune response factors and cellular autophagy pathway	In vivo study in APP/PS1 mice through analysis of DEGs
Kim et al., 2023 [83]	CBDA	6 μΜ, 3 μL (intrahippocampal injection)	↓ Escape latency by CBDA and Δ^9^-THCA compared to Aβ group (MWM test) ↑ Discrimination index (NOR) ↓ Expression of Aβ in the hippocampus ↓ Expression of p-TAU in the hippocampus	In vivo experiments in ICR mice after injection of Aβ_1–42_
	Δ^9^-THCA	12 μΜ, 3 μL (intrahippocampal injection)		
Long et al., 2021 [84]	NlTyr	60 mg/kg p.o.	Increased time spent on the rod (RRT test) Motor coordination enhancement ↓ Escape latency (MWM test) Improvement of cognitive and learning abilities ↓ Aβ_42_ in the CA1 hippocampal region	In vivo experiments in AD APP/PS1 mouse model
Mahdi et al., 2021 [85]	WIN55,212-2	0.5 mg/kg 1 mg/kg 2 mg/kg	Improved escape latency and time spent in target quadrant (MWM test) ↑ SOD, GSH, nestin, GFAP Mitigation of cellular abnormalities in the hippocampus ↓ MDA	In vivo experiments in a AlCl_3_ + D-Gal -treated Wistar rat model
Human studies				
Defrancesco and Hofer, 2020 [86]	Dronabinol drops (Δ^9^-THC)	4.9–6.7 mg/day p.o.	Improvement in emotional state Mitigation of disruptive behaviour, aggression and suppression Absence of ADRs	Case report on a 69-year-old AD patient with severe NPS (depression, paranoid perception)
Alexandri et al., 2023 [87]	CBD oil drops	3% p.o. for 6 months	↓ NPI score Improvement in BPSD	Comparative study in 20 patients with dementia between 3% CBD and usual treatment
Herrmann et al., 2019 [88]	Nabilone	1–2 mg/day p.o.	↓ NPS (NPI-NH) ↓ Anxiety (CMAI) ↓ Malnutrition (MNA-SF) ↑ Cognitive function (sMMSE)	Randomized, double-blind, crossover clinical trial in 39 patients with moderate to severe AD
van den Elsen et al., 2015 [89]	Δ^9^-THC	1.5 mg/3 times/day	Non-statistically significant differences in NPI score, CMAI, Quality of Life-Alzheimer’s Disease, Barthel Index between Δ^9^-THC and placebo groups Well-tolerated treatment with no severe ADRs	Randomized controlled trial in 50 patients with dementia
van den Elsen et al., 2015 [90]	Δ^9^-THC	0.75 mg/2 times/day 1.5 mg/2 times/day	Non-statistically significant differences in NPI score Well-tolerated treatment with no severe ADRs	Randomized controlled trial in 22 patients with dementia and clinically relevant NPS
Palmieri and Vadalà, 2023 [91]	Cannabins extract in oil	1 mL/day (22% Δ^9^-THC, 0.5% CBD) p.o.	↓ Irritability, restlessness, sleep disturbances, apathy (NPI) ↓ Behaviors of physical and verbal aggression (CMAI) ↓ Cognitive impairment from mild to moderate (MMSE)	Limited-size cohort study in 30 patients with moderate-to-severe AD
Ruver-Martins et al., 2022 [32]	Cannabis extract	Microdoses (most often 500 µg p.o.) of 8:1 Δ^9^-THC:CBD extract for 22 months	↑ MMSE ↓ ADAS-COG Stabilization of results with continued treatment	Case report on a 75-year-old patient with mild AD (memory impairment, spatiotemporal disorientation)
van den Elsen et al., 2017 [92]	Δ^9^-THC	1.5 mg/2 times/day for 3 days	↑ Standing sway with eyes closed, trunk sway, stride length No changes in dual-task walking	Randomized controlled trial in 18 patients with dementia

Abbreviations: Aβ: amyloid beta; AD: Alzheimer’s disease; ADAS-COG: Alzheimer’s Disease Assessment Scale—Cognitive Subscale; ADR: adverse drug reaction; 2-AG: 2-arachidonylglycerol; AlCl_3_: aluminum chloride; Bax: Bcl-2-associated X protein; Bcl-2: B cell leukemia/lymphoma 2 protein; BPSD: behavioral and psychological symptoms of dementia; CBD: cannabidiol; CBDA: cannabidiol acid; CB_1_R: cannabinoid receptor 1; CB_2_R: cannabinoid receptor 2; CMAI: Cohen-Mansfield agitation inventory; COX-2: cyclooxygenase-2; DEGs: differentially expressed genes; D-Gal: D-galactosidase; ΔΨm: mitochondrial membrane potential; ER: endoplasmic reticulum; FAAH: fatty acid amide hydrolase; GFAP: glial fibrillary acidic protein; GSH: glutathione; GSK-3β: glycogen synthase kinase-3 beta; ICR: Institute of Cancer Research; IFN-γ: interferon γ; IL-1β: interleukin-1β; IL-6: interleukin-6; iNOS: inducible nitric oxide synthase; i.p.: intraperitoneally; LPS: lipopolysaccharide; LTP: long-term potentiation; MDA: malondialdehyde; MMSE: mini mental state examination; MNA-SF: mini-nutritional assessment short-form; MWM: Morris water maze; NlTyr: N-linoleyltyrosine; NO: nitric oxide; NOR: novel object recognition; NPI: neuropsychiatric inventory; NPI-NH: neuropsychiatric inventory—nursing home; NPS: neuropsychiatric symptoms; PGE-2: prostaglandin E2; p.o.: per os; PPAR-γ: peroxisome proliferator-activated receptor-γ; PSMB5: proteasome subunit beta type-5; p-TAU: phosphorylated TAU; PSEN1: presenilin 1; PUMA: p53 upregulated modulator of apoptosis; ROS: reactive oxygen species; RRT: rotarod test; sMMSE: standardized mini mental state examination; SOD: superoxide dismutase; Δ^9^-THC: tetrahydrocannabinol; Δ^9^-THCA: tetrahydrocannabinolic acid; TNF-a: tumor necrosis factor-a; UPR: unfolded protein response.

**Table 5:** Main results from the studies with non-selective agonists of CB_1_R and CB_2_R associated with cholinergic pathways and molecules that act through pathways related to the ECS.

Reference	Cannabinoid	Dosage	Major Results	Model Used
Non-selective agonists of CB_1_R and CB_2_R associated with cholinergic pathways				
Nuñez-Borque et al., 2020 [93]	NP137	2.5 μΜ 5 μΜ 1 mg/kg/day p.o.	In vitro ↓ Aβ-induced cell death Inhibition of BChE (NP137) and BACE-1/BChE ↓ AD cell proliferative activity, ERK1/2 phosphorylation, AD lymphoblast p21 content In vivo ↓ Escape latency (MWM test) and restoration of cognitive functions (NP137)	In vitro experiments in immortalized lymphocytes of patients with delayed AD and in vivo experiments in TgAPP mice
	NP148	5 μΜ		
Modulator molecules that act through pathways related to the endocannabinoid system				
Balleza-Tapia et al., 2018 [94]	Capsaicin (Trpv-1 agonist)	10 μΜ	Prevention of nerve cell decline Resynchronization of emission of energy potentials in CA3 pyramidal cells Restoration of the balance between excitatory/inhibitory potentials	In vitro experiments in Aβ-treated hippocampal cells
Xiang et al., 2022a [96]	O-1602 (GPR-55 agonist)	2 μg/mouse 4 μg/mouse i.c.v.	↑ Cognitive functions (MWM, NOR tests) ↓ sAβ_1–42_ in hippocampus and frontal cortex ↓ RhoA, ROCK2 Reverse of GPR-55 downregulation ↓ MDA, ↑ GSH, SOD, CAT	In vivo experiments in Aβ_1–42_-treated mice
Xiang et al., 2022b [98]			Improvement in synaptic function (upregulation of PSD-95) ↓ Pro-inflammatory cytokines, microglial activation Prevention of AChE upregulation	In vivo experiments in STZ-treated mice
Wang et al., 2022 [99]			Prevention of hippocampal cell apoptosis (TUNEL staining) ↓ Expression of NF-κB p65, Bax ↓ Caspase-3 activity ↑ Expression of Bcl-2, anti-inflammatory cytokines	In vivo experiments in LPS-treated mice

Abbreviations: Aβ: amyloid beta; AChE: acetylcholinesterase; AD: Alzheimer’s disease; BACE-1: β-site amyloid precursor protein cleaving enzyme; Bax: Bcl-2-associated X protein; BChE: butyrylcholinesterase; Bcl-2: B cell leukemia/lymphoma 2 protein; CAT: catalase; ERK1/2: extracellular signal-regulated kinase 1/2; GPR-55: G-protein coupled receptor 55; GSH: glutathione; i.c.v.: intracerebroventricular; LPS: lipopolysaccharide; MDA: malondialdehyde; MWM: Morris water maze; NF-κB: nuclear factor-κB; NOR: novel object recognition; p.o.: per os; PSD-95: postsynaptic density protein 95; RhoA: Ras homolog family member A; ROCK2: Rho-associated coiled-coil-containing protein kinase 2; SOD: superoxide dismutase; STZ: streptozotocin; Trpv-1: transient receptor potential cation channel subfamily V member 1; TUNEL: terminal deoxynucleotidyl transferase dUTP nick-end labeling.

**Table 6:** Main results from the combination studies with agonists belonging to different classes.

Reference	Cannabinoid	Dosage	Major Results	Model Used
Elmazoglu et al., 2020 [100]	AEA	1–1000 μΜ	↑ Cell viability, ↓ ROS (URB597 > WIN55,212-2 ≈ CP55-940 > 2-AG ≈ AEA) ↓ Aggregation of Aβ (URB597 ≈ AEA > rest of cannabinoids) ↑ SOD, CAT, GPx, GRx and Nrf2	In vitro experiments in rat hippocampal neurons—model of combined toxic hyperglycemia and Aβ_1–42_
	2-AG			
	CP55-940			
	WIN55,212-2			
	URB597 (FAAH inhibitor)			
Schubert et al., 2019 [101]	Δ^8^-THC	250 nΜ–10 μM	Reduction in accumulated Aβ by Δ^8^-THC and Δ^9^-THC CBD, DMCBD, CBGA, Δ^9^-THC suppressed pro-inflammatory response of microglial cells to LPS (EC_50_ < 10 μM) Neuroprotection independent of CB_1_R and CB_2_R activation	In vitro experiments in HT22 and MC65 cells after induction of C99 production
	Δ^9^-THC			
	Δ^9^-THCA			
	CBD			
	CBDA			
	DMCBD			
	CBDV			
	CBG			
	CBGA			
	CBC			
	CBN			
	MCBN			
Navarro-Dorado et al., 2016 [102]	WIN55,212-2	0.2 mg/kg/day p.o.	Combined action Restoration of normal (↓) Col IV levels in anterior cortical positive vessels, ↓ Col IV vascular density Restoration of the vasodilatory action of ACh ↓ Microvascular lesions	In vivo experiments in TgAPP AD mice
	JWH-133			
Stanciu et al., 2024 [103]	JWH-133	0.2 mg/kg p.o. for over 90 days	Combined action Reduced cognitive decline and anxiety-like behavior (NOR and EPM tests) ↓ Size and number of Aβ plaques, cerebral glucose metabolism, mTOR, CB_2_R Enlarged astrocytes ↑ M1 receptors	In vivo experiments in APP/PS1 mice
	Cannabixir^®^ Medium Flos	2.5 mg/kg p.o. for over 90 days		
	Donepezil	0.65 mg/kg p.o.		

Abbreviations: Aβ: amyloid beta; ACh: acetylcholine; AD: Alzheimer’s disease; AEA: anandamide; 2-AG: 2-arachidonylglycerol; CAT: catalase; CBC: cannabichromene; CBD: cannabidiol; CBDA: cannabidiol acid; CBDV: cannabidivarin; CBG: cannabigerol; CBGA: cannabigerolic acid; CBN: cannabinol; CB_1_R: cannabinoid receptor 1; CB_2_R: cannabinoid receptor 2; Col IV: collagen IV; DMCBD: dimethyl cannabidiol; EPM: elevated plus maze; FAAH: fatty acid amide hydrolase; GPx: glutathione peroxidase; GRx: glutaredoxin; LPS: lipopolysaccharide; MCBN: cannabinol methyl ether; mTOR: mammalian target of rapamycin; NOR: novel object recognition; Nrf2: nuclear factor erythroid 2-related factor 2; p.o.: per os; ROS: reactive oxygen species; SOD: superoxide dismutase; Δ^9^-THC: tetrahydrocannabinol; Δ^9^-THCA: tetrahydrocannabinolic acid.

## Abbreviations for All Figures

Aβ: amyloid beta; ACh: acetylcholine; AChE: acetylcholinesterase; ACEA: arachi-donyl-2’-chloroethylamide; ACPA: arachidonylcyclopropylamide; AD: Alzheimer’s disease; ADAS-COG: Alzheimer’s Disease Assessment Scale—Cognitive Subscale; ADR: adverse drug reaction; AEA: anandamide; 2-AG: 2-arachidonylglycerol; Akt: protein kinase B; AlCl_3_: aluminum chloride; BACE-1: β-site amyloid precursor protein cleaving enzyme; Bax: Bcl-2-associated X protein; BChE: butyr-ylcholinesterase; Bcl-2: B cell leukemia/lymphoma 2 protein; BCP: β-caryophyllene; BDNF: brain-derived neurotrophic factor; BPSD: behavioral and psychological symptoms of dementia; CAT: catalase; CBC: cannabichromene; CBD: cannabidiol; CBDA: cannabidiol acid; CBDV: cannabidivarin; CBG: cannabigerol; CBGA: cannabigerolic acid; CBN: cannabinol; CB_1_R: cannabinoid receptor 1; CB_2_R: cannabinoid receptor 2; CeA: central amygdala; CMAI: Cohen-Mansfield agitation inventory; Col IV: collagen IV; COX-2: cyclooxygenase-2; DEGs: differentially expressed genes; D-Gal: D-galactosidase; DMCBD: dimethyl cannabidiol; ΔΨm: mitochondrial membrane potential; e.o.d.: every other day; EPM: elevated plus maze; ER: endoplasmic reticulum; ERK1/2: extracellular signal-regulated kinase 1/2; EV: extra-dimensional; FAAH: fatty acid amide hydrolase; GFAP: glial fibrillary acidic protein; GPx: gluta-thione peroxidase; GPR-55: G-protein coupled receptor 55; GRx: glutaredoxin; GSH: glutathione; GSK-3β: glycogen synthase kinase-3 beta; i.c.v.: intracerebroventricular; IFN-γ: interferon γ; IL-1β: interleukin-1β; IL-6: interleukin-6; iNOS: inducible nitric oxide synthase; i.p.: intraperitoneally; LPS: lipopolysaccharide; LTP: long-term potentiation; MAGL: monoglycerol lipase; MDA: malondialdehyde; miR: microRNA; MMSE: mini mental state examination; MNA-SF: mini-nutritional assessment short-form; MCBN: cannabinol methyl ether; mPFC: medial prefrontal cortex; MWM: Morris water maze; NF-κB: nuclear factor-κB; NlTyr: N-linoleyltyrosine; NO: nitric oxide; NOR: novel object recognition; NPI: neuro-psychiatric inventory; NPI-NH: neuropsychiatric inventory—nursing home; NPS: neuropsychiatric symptoms; Nrf2: nuclear factor erythroid 2-related factor 2; OAE: O-arachidonylethanolamine; OBX: olfactory bulbectomy; o.d.: once daily; OKA: okadaic acid; OLR: object location recognition; PA: passive avoidance; PGE-2: prostaglandin E2; PKA: protein kinase A; p.o.: per os; PPAR-γ: peroxisome proliferator-activated receptor-γ; PSMB5: proteasome subunit beta type-5; p-TAU: phosphorylated TAU; PSEN1: presenilin 1; PSD-95: postsynaptic density protein 95; PUMA: p53 upregulated modulator of apoptosis; REV1: reversal 1; RhoA: Ras homolog family member A; ROCK2: Rho-associated coiled-coil-containing protein kinase 2; ROS: reactive oxygen species; RRT: rotarod test; RVD: (m)RVD-hemopressin; s.c.: subcutaneously; sMMSE: standardized mini mental state examination; SOD: superoxide dismutase; STZ: streptozotocin; Δ^9^-THC: tetrahydrocannabinol; Δ^9^-THCA: tetrahydrocannabinolic acid; TLR4: toll-like receptor 4; TNF-a: tumor necrosis factor-a; TrkB: tropomyosin receptor kinase B; Trpv-1: transient receptor potential cation channel subfamily V member 1; TUNEL: terminal deoxynucleotidyl transferase dUTP nick-end labeling; UPR: unfolded protein response; VD: (m)VD-hemopressin.
